# Supplementary material for: Coherent Charge Transport in Ballistic InSb Nanowire Josephson Junctions
Source: Sci Rep. 2016 Apr 22;6:24822. doi: 10.1038/srep24822 (PMC4840339; doi:10.1038/srep24822)
Supplement: Supplementary Information [file srep24822-s1.pdf]

Supplementary Information for

Coherent charge transport in ballistic InSb nanowire Josephson junctions

S. Li[1], N. Kang[1,*], D. X. Fan[1], L. B. Wang[1], Y. Q. Huang[1], P. Caroff[a),2], and H. Q. Xu[1,3*]

[1] Key Laboratory for the Physics and Chemistry of Nanodevices and Department of Electronics, Peking University, Beijing 100871, China

[2] I.E.M.N., UMR CNRS 8520, Avenue Poincaré, BP 60069, F-59652 Villeneuve d'Ascq, France

[3]Division of Solid State Physics, Lund University, Box 118, S-221 00 Lund, Sweden

[*] Corresponding author: nkang@pku.edu.cn

[*] Corresponding author: hqxu@pku.edu.cn

[a)] Present address: Department of Electronic Materials Engineering, Research School of Physics and Engineering, The Australian National University, Canberra, ACT 0200, Australia

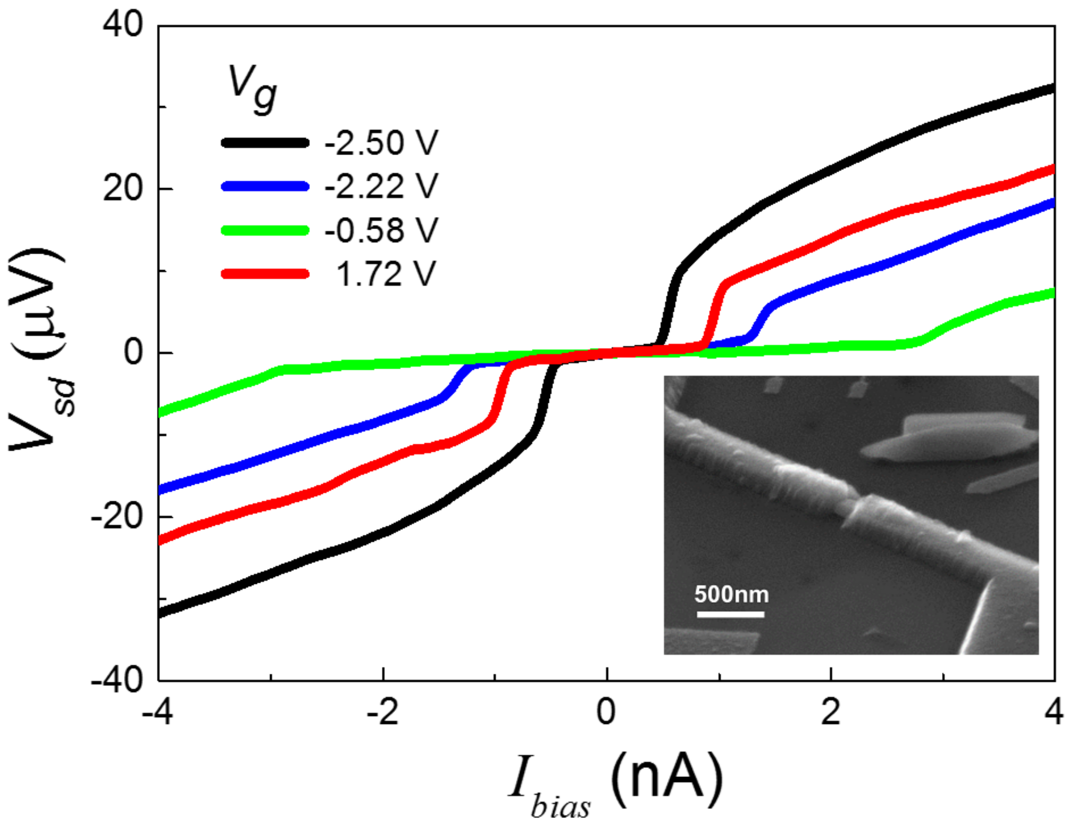

FIG. S1: Measured voltage V_{sd} as a function of bias current I_{bias} for an InSb nanowire-based Josephson junction device (i.e., device D4) with nanowire diameter D ~ 120 nm and contact separation L ~ 200 nm at T = 10 mK and different gate voltages V_g. Here, a gate-tunable supercurrent is seen. The inset shows an SEM image of the measured device.
